# Invasive Round Goby (*Neogobius melanostomus*) Fish from the Southern Baltic as a Source of Arsenic and Selenium—Food Safety Aspects

**DOI:** 10.3390/foods13111779

**Published:** 2024-06-05

**Authors:** Arkadiusz Nędzarek, Przemysław Czerniejewski

**Affiliations:** 1 Department of Aquatic Bioengineering and Aquaculture, Faculty of Food Sciences and Fisheries, West Pomeranian University of Technology in Szczecin, K. Królewicza 4, 71-550 Szczecin, Poland; 2 Department of Commodity, Quality Assessment, Process Engineering and Human Nutrition, Faculty of Food Sciences and Fisheries, West Pomeranian University of Technology in Szczecin, K. Królewicza 4, 71-550 Szczecin, Poland; pczerniejewski@zut.edu.pl

**Keywords:** dietary reference, estimated daily intake, carcinogenic risk, food analysis, food composition

## Abstract

Minimizing human exposure to arsenic (As) and ensuring an adequate dietary intake of selenium (Se) are significant issues in research on food sources. This study measured the content of As and Se in the muscles, gills, liver, and gonads of the fish round goby (*Neogobius melanostomus*) to assess the benefits and risks associated with their consumption. This was achieved by using dietary reference intake (DRI), estimated daily intake (EDI), target hazard quotient (THQ), and carcinogenic risk (CR). The elements were analyzed by atomic absorption spectrometry. The mean concentrations of As and Se (in μg kg^−1^ wet weight) were 25.1 and 161.4 in muscle, 58.8 and 367.4 in liver, 47.4 and 635.3 in gonads, and 16.4 and 228.5 in gills, respectively. Arsenic in the muscle portion of fish accounted for up to 0.5% of the DRI, while Se constituted approximately 30% of the DRI. The EDI values were below the reference oral dose (RfD). The THQ were much below the permissible levels (THQ < 1), and the CR were at least within the permissible limit (CR < 10^−4^). With regard to the As content, round goby muscles can be deemed safe for consumers. They may also be a valuable source of Se in the human diet. However, round goby consumption should be monitored for the proper and safe intake of these elements.

## 1. Introduction

Arsenic (As) and selenium (Se) are metalloids with highly interesting bioactive properties [1,2]. This is associated with their ambivalent effect on living organisms. On one hand, they exhibit specific toxicity and potential carcinogenicity (especially As), and on the other hand, they have positive impact, especially Se [3,4,5]. Research on their effects on the human body yields conflicting results [6,7,8].

Despite its various applications in medicine, industry, and agriculture, As is a well-known carcinogenic agent [9]. High concentrations of inorganic As are hazardous to human health, but chronic exposure to low levels of As in food can also lead to As poisoning and an increased risk of cancer [10]. As-related cancers and metabolic diseases may have a long latency period, and many patients exposed to As remain asymptomatic for years [7].

Currently, Se is recognized as an important trace element in the human diet. It is a component of selenoproteins, including enzymatically active ones such as selenocysteine, which participates in redox reactions [11]. However, the range between necessary dietary doses and toxic doses is relatively narrow [1]. Low doses of Se and selenoproteins can induce dermatological, endocrinological, and neurological changes [8]. Conversely, excessive exposure to Se can facilitate the development of chronic degenerative diseases [6]. In addition, Se has a positive impact on the detoxification of Hg and As, as an antagonist alleviating toxicity symptoms caused by these neurotoxins [12,13,14].

Arsenic and selenium are naturally occurring elements that are commonly found in the environment, particularly in rocks and soil [8]. Currently, significant sources of contamination in terrestrial and aquatic ecosystems include industrial activities such as mining, the use of artificial fertilizers, the electrical industry, and mineral combustion [8,10,15]. Both elements occur in inorganic forms as well as in organic compounds. [2,10,16]. In the case of As, inorganic forms (especially the gaseous form) are considered more toxic than organic forms [6,10]. Fish [17] and humans [18] absorb organic forms of Se more readily than inorganic Se.

Fish exhibit a very wide range of concentrations of As and Se (comparative summary in Table 1). The bioaccumulation of As and Se in fish is influenced by a number of factors, including the concentration of these elements in the environment, their speciation, and the fish species [16,17]. The data indicate a need for the continuous monitoring of As and Se concentrations in fish from different fishing areas and in different species. This is particularly pertinent in light of the increasing global consumption of fish [19], which, despite its high nutritional value (high-quality protein, omega-3 fatty acids, vitamins, and minerals), may result in an increased intake of hazardous compounds, including trace elements such as Hg or As [2,12]. Monitoring the levels of As and Se in fish is also crucial due to the accumulated and prolonged intake of these elements, which can lead to adverse health effects. This kind of research is also important because legal regulations and recommendations regarding the control of exposure to As and Se are still under discussion and being updated [4,5,8,20].

For example, the Joint FAO/WHO Expert Committee on Food Additives recommended a provisional tolerable weekly intake (PTWI) for inorganic As at the level of 15 μg kg^−1^ body weight. However, this recommendation was subsequently withdrawn in 2011 [21]. This level is also not recommended by the EFSA Panel on Contaminants in the Food Chain (CONTAM), which, in 2009, adopted a scientific opinion indicating that the minimum amount of inorganic As causing a clear, low-risk to health falls within the range of 0.3 μg kg^−1^ to 8 μg kg^−1^ body weight per day [22]. Subsequent reports have corroborated this opinion, indicating that even lower inorganic As consumption does not preclude the possibility of risk to selected consumers [5,20].

**Table 1:** Comparison of total arsenic and selenium concentrations (in mg kg^−1^) in the muscles of different fish species, divided into freshwater and marine fish.

Area (Reference)	Species	Se	Area (Reference)	Species	As	
Bovan Reservoir, Serbia [23]	Pikeperch (*Sander lucioperca*)	0.091	Bovan Reservoir, Serbia [23]	Pikeperch (*Sander lucioperca*)	0.003	Fresh water
	Catfish (*Silurus glanis*)	0.142		Catfish (*Silurus glanis*)	0.002	
	Pike (*Esox lucius*)	0.170		Pike (*Esox lucius*)	0.001	
	Prussian carp (*Carassius gibelio*)	0.237		Prussian carp (*Carassius gibelio*)	0.003	
	Bream (*Abramis brama*)	0.258		Bream (*Abramis brama*)	0.002	
Colorado River, USA [24]	Common carp (*Cyprinus carpio*)	6.03	Tercan Dam Lake, Turkey [25]	Common carp (*Cyprinus carpio*)	0.383	
	Fathead minnow (*Pimephales promelas*)	11.89		Bulatmai barbell (*Luciobarbus capito*)	0.124	
	Pike (*Esox lucius*)	2.53		Tigris scraper (*Capoeta umbla*)	0.186	
	Black bullhead (*Ameiurus melas*)	4.35		Grass carp (*Ctenopharyngodon idella*)	0.774	
	Yellow perch (*Perca flavescens*)	13.53	French fishing areas, France [26]	Pikeperch (*Sander lucioperca*)	0.029–0.233	
	Rainbow trout (*Oncorhynchus mykiss*)	3.38		Bream (*Abramis brama*)	0.029–0.331	
				Pike (*Esox lucius*)	0.017–0.356	
Mediterranean Sea [12]	Blue shark (*Prionace glauca*)	0.63	Bay of Bengal, Bangladesh [27]	Gold-spotted grenadier anchovy (*Coilia dussumieri*)	1.330	Sea
	Porbeagle (*Lamna nasus*)	1.25				
	Picked dogfish (*Squalus acanthias*)	1.25		Fringescale sardinella (*Sardinella fimbriata*)	3.930	
	Smooth-hound (*Mustelus mustelus*)	1.03				
	Thornback ray (*Raja clavata*)	0.65	Back Bay, Canada [28]	Whitefish (*Gadus morhua*)	0.77	
				Longnose sucker (*Catostomus catostomus*)	1.15	
Pacific, Hawaii [29]	Yellowfin tuna (*Thunnus albacares*)	1.25	Baltic Sea [30]	Baltic cod (*Gadus morhua*)	0.390	
	Striped marlin (*Tetrapturus audax*)	0.72		European sprat (*Sprattus sprattus*)	0.636	
	Blue marlin (*Makaira nigricans*)	1.59		Baltic herring (*Clupea harengus membras*)	0.460	
	Swordfish (*Xiphias gladius*)	0.39		European flounder (*Platichthys flesus*)	0.588	
Central Pacific [31]	Mako shark (*Isurus oxyrinchus*)	0.32	Hai Phong, Vietnam [32]	Northern snakehead (*Channa argus*)	1.180	
	Swordfish (*Xiphias gladius*)	0.43		Iridescent shark catfish (*Pangasianodon hypophthalmus*)	1.660	
	Skipjack tuna (*Katsuwonus pelamis*)	1.56				
Mediterranean Sea [33]	European pilchard (*Sardina pilchardus*)	0.15	Gulf of Guinea [34]	Madeiran sardinella (*Madeiran sardinella*)	1.560	
	European hake (*Merluccius merluccius*)	0.29		Angola dentex (*Dentex angolensis*)	1.870	
	European seabass (*Dicentrarchus labrax*)	0.30		European barracuda (*Sphyraena sphyraena*)	0.820	

The EFSA Panel [4] has lowered the tolerable upper intake level (UL) for adults from 400 μg daily to 255 μg daily due to concerns related to the negative effects of excessive Se consumption [35]. This was based on the observed lowest observed adverse effect level (LOAEL) at an intake of 330 μg Se per day. The EFSA Panel [4] additionally recommends monitoring Se intake from supplements.

As indicated in Table 1, total As and Se are measured in various freshwater and marine fish species. However, to our knowledge, such studies have not yet been conducted for round goby. Knowing the concentrations of As and Se in this species can provide a valuable foundation for planning its use for human consumption, especially since it is one of the most widespread invasive fish species in the Baltic Sea [36]. Currently, commercial fishing for round goby occurs in the Baltic Sea off the coasts of Lithuania and Latvia [37], where they are sold as fresh fish or processed canned products [38]. Given the continued spread of round goby in Europe [36,39] and the high nutritional value and sensory quality of its meat [37], increasing the catching of this fish in other parts of the Baltic for food production could be an excellent strategy for managing their populations. This approach complies with Regulation (EU) No 1143/2014 [40], which prohibits the reintroduction of invasive fish species into water bodies after fishing.

In light of the aforementioned considerations, (i) the content of total As and total Se was examined for the first time in invasive round goby (*Neogobius melanostomus*) from the waters of the Southern Baltic; (ii) the accumulation of both metalloids in muscles, gills, gonads, and liver was compared between the water bodies; and (iii) consumer risk was assessed by calculating dietary reference intakes (DRIs), estimated daily intake (EDI), carcinogenic risk (CR), and target hazard quotient (THQ). The health risk associated with the consumption of As was calculated for its concentration representing 10% of the total As measured, which, according to [2,7], corresponds to the approximate proportion of inorganic As in total As.

## 2. Methods

### 2.1. Study Area

**Figure 1:**
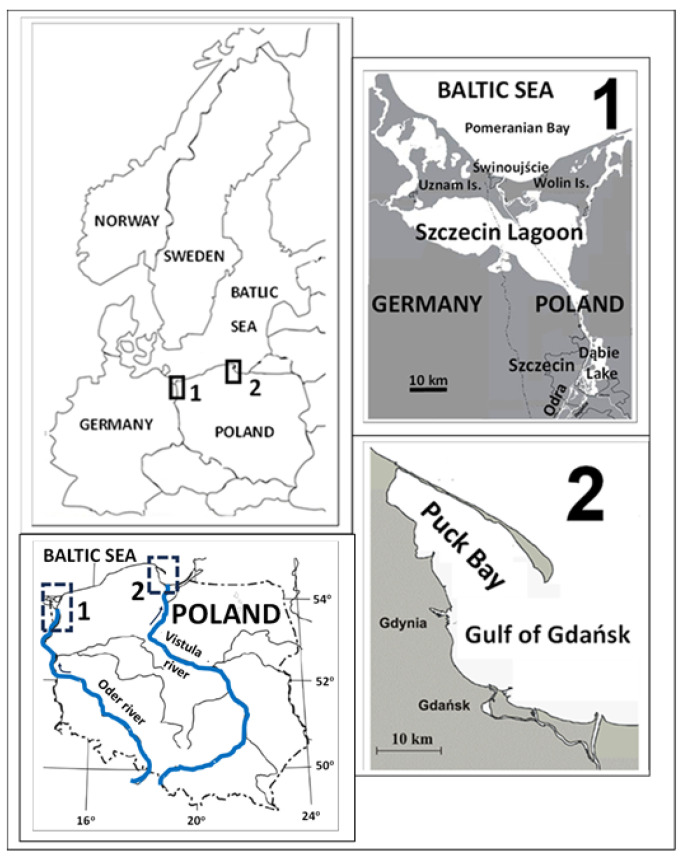
Study areas (1—Dąbie Lake and Szczecin Lagoon; 2—Puck Bay).

Water, bottom sediment, and fish samples were collected from Dąbie Lake, Szczecin Lagoon, and Puck Bay, which are located in the southern part of the Baltic Sea basin (Figure 1). These water bodies share relatively shallow average depth and high anthropogenic pressure but differ in the influence of marine and freshwater on their chemical conditions.

Dąbie Lake (area of 56 km^2^, average depth of 2.61 m, maximum depth of 7 m) is situated within the city limits of Szczecin. The Eastern Oder River flows into it from the south, and to the north, it connects through a wide channel with the southern end of Szczecin Lagoon. The lake’s waters are subject to considerable pressure from the Oder River waters, as well as from chemical plants and the shipbuilding industry [41,42].

Szczecin Lagoon (area of 687 km^2^, average depth of 3.8 m, maximum depth of 8.5 m) is primarily fed by the waters of the Oder River. The water body is brackish, with salinity in the central part ranging between 0.5 PSU and 2 PSU. Periodic intrusions of Baltic Sea water (6 PSU salinity) mainly occur via the Świna Channel [43].

Puck Bay (area of 364 km^2^) forms the northwestern part of the Gulf of Gdańsk (about 1.4% of its area) and is divided into a shallow inner part (average depth of 3.1 m, maximum 9.4 m) and an outer part (average depth of 20.5 m, maximum 54 m). The salinity of water in this area ranges from 7.31 PSU to 7.65 PSU. The hydrological and hydrochemical conditions are mainly influenced by saline water inflows from the Gulf of Gdańsk, into which the Vistula River flows (the largest river in the region, with an average flow of more than 1000 m^3^ s^−1^) [44].

### 2.2. Fish, Water, and Bottom Sediments Sample Collection

The fish for the study were collected from fisheries in June and July 2022. A total of 16 fish (8 females and 8 males) were taken from each water body.

At the same time, three water samples were collected from each reservoir at a depth of 0.5 m in the fishing area using a Ruttner-type apparatus. Additionally, four bottom sediment samples were collected using a 250 cm^3^ van Veen scoop.

### 2.3. Dissection of Sampled Fish

Immediately after capture, the fish were weighed on an Axis 2000 electronic balance (with an accuracy of ±0.1 g), and their total length (TL) and standard length (SL) were measured using an electronic caliper (with an accuracy of ±0.1 mm). The gills, gonads, liver, and muscle were then removed from each fish and frozen at −20 °C until As and Se content could be determined [45].

### 2.4. Elemental Analysis

The determination of As and Se in water, sediments, and round goby was carried out according to the methodologies described by APHA [46].

Water samples were digested with HNO_3_ at a volume ratio of 10:1 at 100 °C. Bottom sediments, after drying to a constant weight at 90 °C, were sieved through a 2 mm sieve. The bottom sediment fraction was then digested with concentrated HNO_3_ at a ratio of 5 g dry weight of sediment to 10 mL of HNO_3_. The digestion time was 30 min, and the temperature was 200 °C. The solution obtained was then diluted to 25 mL.

Muscle, gill, and liver samples (1.00 ± 0.01 g wet weight) and gonad samples (0.1 ± 0.01 g wet weight) were digested in 6.0 mL of a mixture of HNO_3_ and HClO_4_ (volume ratio 5:1). After digestion, the samples were diluted with water to a total volume of 25 mL. The samples were digested in a high-pressure microwave digester (Speedwave Xpert, Berghof, Eningen, Germany). The digestion process was conducted using Ultrapure-concentrated HNO_3_ and HClO_4_ (Merck, Darmstadt, Germany) and Milli-Q water (18.2 MΩ).

Arsenic and Se were determined by graphite furnace atomic absorption (GFFA) using a Hitachi ZA3000 series polarized Zeeman atomic absorption spectrometer (Hitachi High Technologies Corporation, Tokyo, Japan). Calibration curves were established using certified standard solutions (1000 mg L^−1^) from Merck (Germany).

### 2.5. Quality Control and Quality Assurance

To ascertain the absence of contamination in the reagents, reagent blanks were employed. A spike-and-recovery test was conducted on a random selection of water, bottom sediment, and fish samples to validate the accuracy and precision of the analytical method and digestion. The accuracy of the analytical method was tested using the dogfish liver NCR-DOLT-5 reference material (National Research Council Canada). Elemental recoveries were 91% for As and 94% for Se.

### 2.6. Estimated Daily Intake

The estimated daily intake (EDI; in mg kg^−1^ day^−1^) of elements from the consumption of fish muscle was calculated using the following equation [47]:

EDI = (Ci × IR)/BW(1)

where Ci is the element concentration in fish muscle (mg kg^−1^ wet weight); IR is the fish muscle intake rate (0.03 kg day^−1^); and BW is the average body weight (70 kg for adults).

The resulting EDI values were compared with the reference (safe) oral dose of the element (RfD). According to the NYS DOH [48], if the ratio of the EDI value of an element to the RfD value is equal to or less than RfD, the risk is minimal; if it is 1–5 times greater than the RfD, the risk is low; if it is 5–10 times greater than the RfD, the risk is moderate; if it is 10 times greater than the RfD, the risk is high.

### 2.7. Nutritional Quality

To evaluate the nutritional quality associated with the consumption of fish muscle, the intake of elements was calculated on the basis of a 100 g wet-weight ratio of muscle using a procedure similar to that described by Nędzarek et al. [49]. Based on the guidelines of EFSA [4], FAO WHO [21], Otten et al. [35], Baars et al. [50], and Salahinejad and Aflaki [51], percentages for individual elements were calculated for the following dietary reference intakes (DRIs):

- Tolerable daily intake (TDI), provisional tolerable weekly intake (PTWI), and acceptable daily intake (ADI) for inorganic As;
- Recommended dietary allowance (RDA) and tolerable upper intake level (UL) for Se.

### 2.8. Human Health Risk Assessment

The assessment of risk associated with the consumption of inorganic As and Se in fish muscle followed the procedure of Tan et al. [52] and the US EPA [53,54]. The non-carcinogenic target hazard quotient (THQ) was calculated using the equation:

THQ = [(EF × ED × IR × Ci)/(RfD × BW × AT)] × 10^−3^ (2)

where EF is the exposure frequency (365 days year^−1^); ED is the exposure duration equivalent to the average human lifetime (70 years); IR is the fish muscle intake rate (30 g day^−1^); Ci is the element concentration in the fish muscle (mg kg^−1^); RfD is the oral reference dose for the contaminant (mg kg^−1^ day^−1^); BW is the average body weight (70 kg for adults); and AT is the exposure time for non-carcinogens (365 days year^−1^ ED).

The THQ represents the ratio of the exposure level to a substance over a specified period of time to the reference dose (RfD) for that particular substance. Thus, THQ ≥ 1 indicates potential health hazards associated with the consumption of certain foods.

The carcinogenic risk (CR) was calculated for inorganic As using the formula

CR = [(EF × ED × IR × Ci × CSF)/(BW × AT)] × 10^−3^ (3)

where the parameters were defined as in Formula (2), except for CSF, which is the cancer slope factor set by the US EPA [54]. According to the US EPA [53], a lifetime cancer risk below 1 × 10^−6^ is negligible, a risk above 1 × 10^−4^ is unacceptable, and a range between 1 × 10^−6^ and 1 × 10^−4^ is acceptable.

For the EDI and THQ calculations, we used the average concentration of inorganic As and Se in each of the compared fish muscle samples.

### 2.9. Statistical Analysis

The statistical difference between the detected heavy metals of water, bottom sediments, and different fish organs (gills, muscles, liver and gonads) was evaluated by one-way ANOVA using Statistica v13.3 software from TIBCO Software Inc. (Palo Alto, CA, USA). Significance of differences (*p* < 0.05) was verified by Tukey’s post hoc test. Pearson’s correlation coefficient (r) was also used. In addition, Principal Component Analysis (PCA) was used to identify the underlying components in the data and to assess the association between the metals. Biological characteristics of females and males were compared using the Mann–Whitney U test.

## 3. Results

### 3.1. Arsenic and Selenium in Water and Bottom Sediments

The average concentrations of total As and Se in the water of the study sites were 0.653 μg L^−1^ and 1.71 μg L^−1^, respectively. At the same time, variations in total As concentrations between water bodies were significant (*p* < 0.05), with Puck Bay having the lowest As (0.307 μg L^−1^) and Dąbie Lake having the highest (0.947 μg L^−1^). No such variation was observed for Se (Table 2).

The mean concentrations of total As and Se in the sediments were 0.383 mg kg^−1^ and 268 mg kg^−1^, respectively. Significantly, the lowest concentrations of both metalloids were observed in sediments from Puck Bay, and the highest in sediments from Dąbie Lake (Table 2).

**Figure 2:**
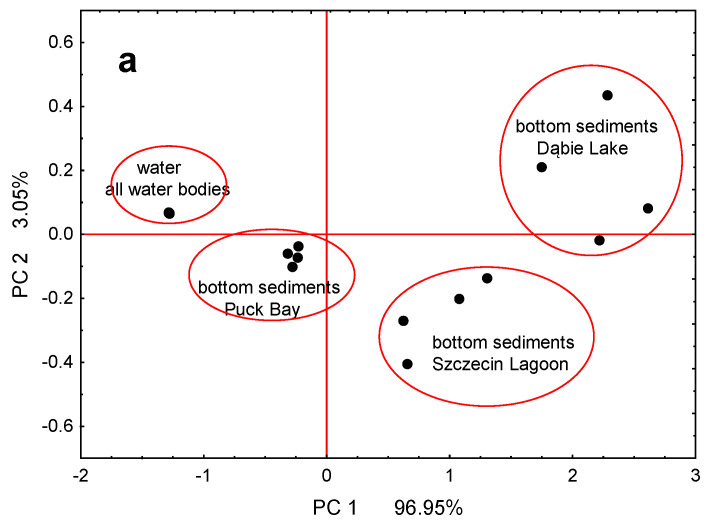

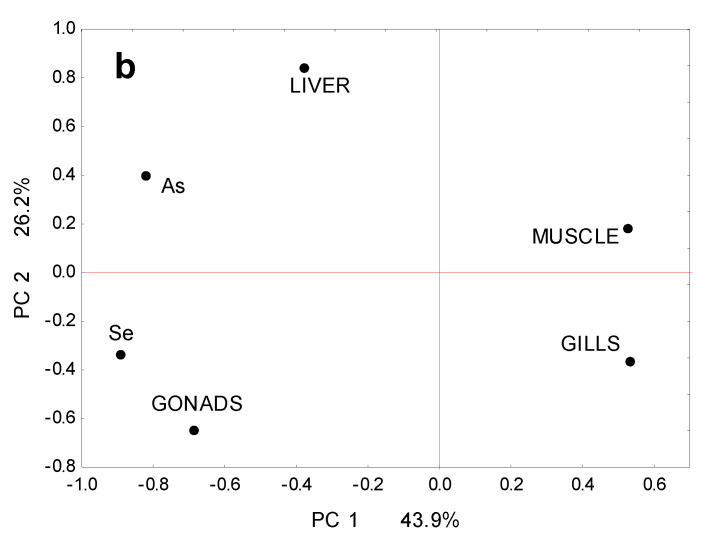
Results of PCA analysis showing the variation in water bodies due to As and Se content in water and bottom sediments (**a**) and the variation of the examined round goby body parts (**b**).

The effect of variation in As and Se content in water and bottom sediments is graphically depicted in Figure 2a. The water bodies formed three separate groups based on the chemical composition of the bottom sediments and one group based on the As and Se contents in the water.

### 3.2. Fish

The mean length of the round goby was 15.82 cm, with a mean weight of 68.54 g (Table 3). There was no significant difference in the length of fish from different water bodies (*p* > 0.05), but fish from Puck Bay had a lower mean weight compared to fish from other water bodies.

In general, females were found to have a smaller total length and weigh less than males. However, these differences were not statistically significant (*p* > 0.05). Notably, only female round gobies from Puck Bay exhibited a significantly greater body weight and total length than males (*p* < 0.05).

### 3.3. Arsenic and Selenium in Round Goby Tissue

Table 4 presents a comparison of As and Se concentrations in the muscles, liver, gills, and gonads of round goby. Given the lack of significant differences (*p* > 0.05) in this comparison, gender division was not considered.

Individual body parts of round goby significantly (*p* < 0.05) differed in As and Se content, and the accumulation series for both elements were different. For As, the series was as follows (average concentrations in μg kg^−1^): liver (58.8) > gonads (47.4) > muscle (25.1) > gills (16.4); for Se, it was gonads (635.3) > liver (367.4) > gills (228.5) > muscle (161.4) (Table 4).

The round goby from Dąbie Lake exhibited the highest concentrations of As and Se in all examined body parts. The highest concentrations of As were found in muscles, liver, and gills, while the highest concentrations of Se were found in gills. Conversely, the lowest concentrations of elements were primarily observed in round goby from Puck Bay (with the exception of Se in gills, where its accumulation was marginally lower than the concentration in the gills of fish from Dąbie Lake) (Table 4).

**Figure 3:**
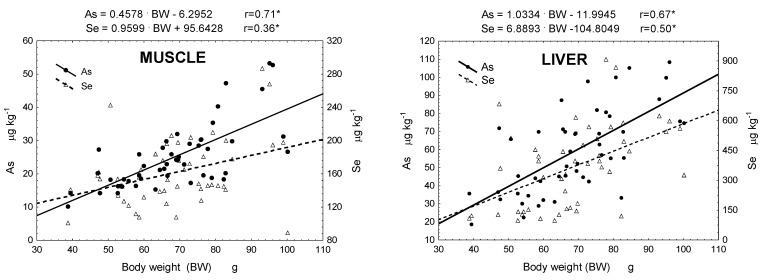

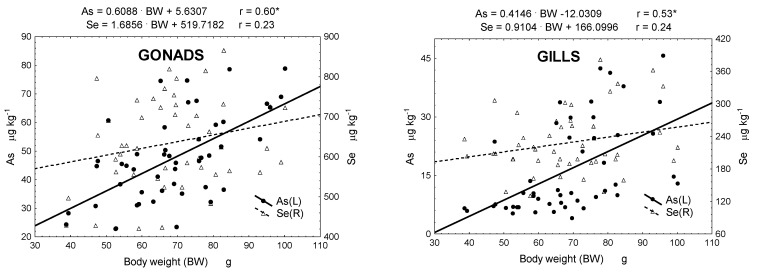
Linear regressions and trend line equations of the correlation between total As and Se in muscle, liver, gonads, and gills and round goby weight (*—statistically significant Pearson r correlations, α = 0.05).

Pearson’s linear regression analysis revealed significant proportional correlations between total As and Se concentrations in muscles and liver and the body weight of round goby. However, in the case of gonads and gills, only total As concentrations exhibited a positive correlation with the weight of fish (r = 0.60 and r = 0.53, respectively) (Figure 3).

### 3.4. Nutritional Quality and Potential Risks to Consumers

Table 5 presents a comparison (values in %) of inorganic As and Se content in a 100 g portion of round goby muscles with reference values for dietary intake (DRI) for adults above 19 years old. The concentrations of inorganic As in the fish muscle portion were below the reference values, averaging 0.17% TWI, 0.20% ADI, and 0.36% TDI. The highest values were observed for round goby from Dąbie Lake, while the lowest were from Puck Bay (Table 5).

The Se content in the round goby muscle portion averaged 29.4% RDA and did not exceed the reference UL values (average of 4.03% UL or 6.33% UL, depending on the applied reference value). The observed values were slightly higher for fish from Dąbie Lake and lower for fish from Puck Bay (Table 5).

Table 6 presents the results of the analysis of human health risks associated with the consumption of inorganic As and Se in a 30 g portion of round goby muscles (portion calculated based on the annual fish consumption in Poland, which was estimated at approximately 12 kg per capita by FAO UN [19]).

The estimated daily intake (EDI, in mg kg^−1^ day^−1^) of As and Se averaged 1.08 × 10^−5^ (for inorganic As) and 6.92 × 10^−5^ (for Se). EDI values were less than 5% of RfD for As and less than 1.5% RfD for Se. Fish from Dąbie Lake exhibited the highest values of these indicators (Table 6).

The target hazard quotient (THQ) averaged 3.59 × 10^−2^ (for inorganic As) and 1.38 × 10^−2^ (for Se), with the highest values observed in the consumption of fish from Dąbie Lake and the lowest in the consumption of fish from Puck Bay. THQ on average exceeded the reference dose (RfD) by two times (Table 6).

The carcinogen risk (CR) was calculated based on the assumption that inorganic arsenic (As) constituted 10% of the total arsenic (As) content in fish muscles, as proposed by Chandel et al. [2], Ro et al. [7], and Tanamal et al. [55]. The lowest carcinogen risk (CR) value (in mg kg^−1^ day^−1^) was determined for fish from Puck Bay (1.61 × 10^−6^), and the highest was for fish from Dąbie Lake (2.23 × 10^−6^) (Table 6).

## 4. Discussion

### 4.1. Water and Bottom Sediment Quality Assessment

The concentration of As in surface waters is typically low, with a range of 0.003 μg L^−1^ to 4.4 μg L^−1^. Much higher concentrations (even exceeding 1000 μg L^−1^) have been recorded in water bodies affected by anthropogenic pollution [56]. For instance, Helios-Rybicka et al. [57] (2005) observed As concentrations in the Oder River ranging from 0.1 μg L^−1^ to 8.75 μg L^−1^, with a decreasing trend downstream. In the southern Baltic watershed, high concentrations of hydrochemical indicators have been observed in the upper reaches of the Oder and Vistula Rivers, where numerous facilities associated with mineral extraction and processing are located. These concentrations decrease towards the river mouths [58].

Similarly, Se concentrations in surface waters are low, ranging from 0.01 μg L^−1^ to 0.35 μg L^−1^ [59]. However, under anthropogenic influence, they can rise above 100 μg L^−1^ [60]. The Se concentrations found in the bodies of water in this study were higher than those recorded in the open waters of the southern Baltic Sea and the Gulf of Gdańsk. In the study by Pałka et al. [61], the median Se concentrations in these two areas were 0.55 μg L^−1^ and 0.25 μg L^−1^, respectively.

Higher concentrations of As and Se in bottom sediments compared to the water in these water bodies are characteristic of aquatic ecosystems. These elements are deposited in bottom sediments through a series of processes, including precipitation, adsorption, chelation with organic compounds, and subsequent sedimentation [56,62].

The elevated concentrations of As and Se in water and bottom sediments in Dąbie Lake (14°39′35.8″ E, 53°24′49.8″ N) and Szczecin Lagoon (14°26′47.9″ E, 53°50′35.3′ N), followed by Puck Bay (18°42′22.3″ E, 54°41′2.5″ N), could be attributed to numerous local sources such as chemical plants, sea and yacht ports, and shipyards [41,42]. For example, As is used in the protective coatings of ships and wood preservation, resulting in sediment concentrations in the Oder estuary that range from approximately 1 mg kg^−1^ to over 40 mg kg^−1^ [63]. This local character of As sources for the Oder Estuary is corroborated by the lower As concentrations in the water and sediments of the Oder River upstream of the studied water bodies [57]. The higher affinity of As and Se for organic matter and the clay fraction of bottom sediments may also contribute to their higher concentrations in Dąbie Lake and Szczecin Lagoon compared to Puck Bay [56]. The former two water bodies are richer in organic matter and clay, while Puck Bay has sandy sediments and is subject to constant, intense water exchange with the Gulf of Gdańsk [64]. These factors may provide a physical explanation for the differences shown in our PCA analysis (Figure 2a). Dąbie Lake, in particular, experiences significant anthropogenic pressure, serving as a reservoir for pollutants transported by the waters of the Oder River and being directly influenced by the Szczecin agglomeration [41,42]. Szczecin Lagoon also faces high anthropogenic pressure, being a third-order estuary of the Oder River, simultaneously influenced by Oder River waters and periodically by Baltic Sea waters. In contrast, Puck Bay, through the Gulf of Gdańsk, is mainly influenced by less polluted marine waters of the Baltic Sea (e.g., Dybowski et al. [44]).

### 4.2. Analysis of As and Se Content in Round Goby

The available data on the As and Se content in round goby from the southern Baltic waters and other regions are limited. To the best of our knowledge, only Subotić et al. [65] investigated total As concentration in whole individuals of two goby species (*Neogobius gymnotrachelus*, *N. melanostomus*) collected from the Danube River (Belgrade section). The results of that investigation ranged from 0.4 μg g^−1^ to 0.95 μg g^−1^ wet weight. The authors proposed that the elevated total As concentrations in gobies could be a consequence of the elevated level of As in the groundwater of Vojvodina and the Pannonian Basin [65]. The results of Subotić et al. [62] and the data presented in Table 1 indicate that the round goby from the southern Baltic had generally lower concentrations of total As and comparable concentrations of Se compared to those recorded in the muscles of other fish species, both freshwater and marine. This can be attributed to the relatively low contamination of the studied water bodies with As [57,58] and the higher bioaccumulation of Se in fish [12,15].

The observed higher concentration of Se than total As is typical for fish (e.g., Milošković and Simić [23]). This is due to the physiological function of Se, which, at the appropriate level, supports the growth and development of fish, enhances the antioxidant capabilities of the organism, and supports immune functions [66]. Significantly higher Se accumulation rates in gonads and liver (metabolically highly active organs) compared to other tissues and organs have also been observed in previous studies [17,67,68]. Similarly, high bioaccumulation of the highly toxic As is noted in the liver of fish, an organ where detoxification occurs, followed by the gills (absorption of As from the water phase), and the lowest in the muscles (which do not actively participate in detoxification processes) [69,70]. These physiological and metabolic functions of the investigated parts of round goby’s body may provide a physical explanation for the demonstrated differences in the PCA plot in our study (Figure 2b).

The observed differences in As and Se concentrations in round goby based on the water body can be attributed to the higher content of these elements (especially As) in Dąbie Lake and Szczecin Lagoon waters, which results in the increased bioavailability of As and Se for fish living there. The relationship between the increased concentrations of As and heavy metals in various fish species and increased contamination by these elements has been documented by Noël et al. [26]. Additionally, Moges et al. [68] demonstrated an increase in Se bioaccumulation in Nile tilapia (*Oreochromis niloticus*) with increasing Se concentration in water. Based on these data, it can also be assumed that round goby exhibits a characteristic ability of fish to bioaccumulate essential and non-essential trace elements, even when their concentrations are low. The bioaccumulation of trace elements, especially non-essential ones (e.g., As), is also proportional to their concentration in the environment. This can be evidenced by the increase in Pb concentrations in round goby from the Gulf of Gorgan (Caspian Sea, Iran) with increasing environmental pollution by this metal [71].

The demonstrated proportional correlation between total As and Se concentrations in the investigated body parts and the body weight of round goby was consistent with the frequently observed relationship in fish, where the bioaccumulation of both essential and non-essential trace elements increases with age, and consequently, with size and weight (e.g., Canpolat et al. [72]). However, as noted by Kaçar [70], there are is clear and consistent relationship between the content of trace elements in individual body parts and the size of fish. Our studies partially confirmed this as correlations were not always significant.

### 4.3. Nutritional Quality and Health Risk Assessment

The majority of As (80–98%) in fish is present in organic compounds (e.g., arsenobetaine, arsenocholine, arsenosugars). They are rapidly eliminated from the human body, making them less significant in terms of food safety. However, the remaining amount of As consists of inorganic derivatives that can have toxic effects [48]. As the maximum limit of inorganic As in fish is not yet established, we compared its content in the muscles of round goby with the dietary reference intakes. The results for all samples represented a maximum of 0.5% of the tolerated inorganic As intake (see Table 5). Our study also indicates that the consumption of 100 g of round goby muscles will not exceed the benchmark dose lower confidence limit (BMDL) introduced by FAO WHO [21] instead of the provisional tolerable weekly intake (PTWI). The BMDL value was established in the range of 2.0–7.0 μg kg^−1^ body weight per day based on the estimated total exposure with diet. The committee noted that the PTWI of 15 μg kg^−1^ body weight (2.1 μg kg^−1^ body weight per day) falls within the BMDL_0.5_ range and is therefore no longer appropriate.

The intake of inorganic As from a portion of round goby muscles also does not exceed the daily inorganic As intake values for European residents. EFSA [22] studies showed that it ranges from 0.13 to 0.59 μg kg^−1^ body weight. This means that an adult with a body weight of 70 kg consumes As daily in the range of 9.1 µg to 71.3 µg. In a subsequent health risk assessment due to inorganic As intake published by EFSA in 2024, this range was established at 0.07–0.33 μg kg^−1^ body weight. It was also noted that even lower exposure to inorganic As raises health concerns [5]. The estimated daily intake (EDI) of inorganic As was also below the reference oral dose (RfD) set by the US EPA [54] at the level of 3.00 × 10^−4^ mg kg^−1^ day^−1^ (EDI did not exceed 5% RfD). Additionally, the target hazard quotient (THQ) can be considered low, as the value of this parameter was below the permissible level (THQ < 1), and the cancer risk can be deemed acceptable, as it was at the level of CR = 10^−6^ [52]. However, it should be noted that the toxic effects of inorganic As are long-lasting and can be dangerous even at low concentrations [5,10,21].

Se is an essential trace element, but due to the narrow range of concentrations at which both positive and negative effects are observed for the organism (e.g., Nogueira et al. [6] and Vinceti et al. [8]), the recommended daily intake (RDA) and upper tolerable intake level (UL) have been established [4,35]. Our study demonstrated that a portion of 100 g of round goby muscles can serve as a valuable supplementary source of Se in the human diet, providing approximately 30% of the RDA. At the same time, the upper tolerable intake level of Se was observed to be at most around 6% of the UL, while the estimated daily intake was below the reference oral dose (averaging about 1.4% RfD) set by the US EPA [54]. It is noteworthy that, from the perspective of a well-balanced diet, Se in fish is primarily in selenoorganic compounds [15,16], which are the most bioavailable forms of Se [73], such as selenomethionine—the most dominant form of Se in fish muscles (from 70% to 90%) [16]. Therefore, fish is considered a foodstuff with the highest natural content of bioactive Se that is rapidly absorbed by the human body [66]. For example, the body absorbs over 90% of Se in selenomethionine but only about 50% of Se in sodium selenite [18].

## 5. Summary

The studied water bodies are subject to local anthropogenic pressure (the highest for Dąbie Lake and the lowest for Puck Bay), which results in elevated concentrations of Se compared to other waters in the southern coastal zone of the Baltic Sea. Concentrations of total As are also higher than those recorded in the estuarine waters of the Vistula and Oder Rivers. Higher concentrations of total As and Se in bottom sediments than in water are characteristic of aquatic ecosystems.

In our study, the accumulation of total As and Se differed between the various parts of the round goby’s body and varied depending on the water body. The liver accumulated the most total As, while the gonads accumulated the most Se. Element concentrations were proportional to fish weight, but correlations were not always significant. The highest concentrations of total As and Se were recorded in fish from Dąbie Lake, likely due to the influence of contaminated waters from the Oder River and the presence of chemical plants and shipbuilding industries in the vicinity.

The meat of round goby from the studied waters can be deemed safe for consumers with regard to its inorganic As content. Consumption of a 100 g portion of this fish’s muscle does not exceed the safe levels recommended by EFSA [5], FAO WHO [21], and US EPA [54]. Furthermore, the carcinogenic risk associated with inorganic arsenic exposure is also considered acceptable.

Moreover, the meat of these fish can be a valuable source of Se for humans, as consuming a 100 g portion of round goby muscle can provide approximately 30% of the recommended daily intake of this element.

The research was financed by the Ministry of Science and Higher Education in Poland through a subsidy for the West Pomeranian University of Technology Szczecin, Faculty of Food Sciences and Fisheries.

## Figures and Tables

**Table 2:** Comparative analysis of As and Se (mean ± standard deviation) detected in water and bottom sediments of Dąbie Lake, Szczecin Lagoon, and Puck Bay.

Elements	Water				Bottom Sediments			
	Dąbie Lake μg L^−1^	Szczecin Lagoon	Puck Bay	Average	Dąbie Lake mg kg^−1^	Szczecin Lagoon	Puck Bay	Average
As	0.947 ^B^	0.703 ^B^	0.307 ^A^	0.652	0.675 ^C^	0.322 ^B^	0.153 ^A^	0.383
	±0.075	±0.152	±0.025	±0.293	±0.076	±0.088	±0.012	±0.235
Se	2.00 ^A^	1.73 ^A^	1.41 ^A^	1.71	0.373 ^C^	0.297 ^B^	0.136 ^A^	0.268
	±0.66	±0.32	±0.05	±0.451	±0.055	±0.023	±0.005	±0.108

^A,B,C^—the same indices in the rows (separately for water and bottom sediment samples) indicate no significant differences in metal concentrations at the significance level of *p* < 0.05 (one-way ANOVA, Tukey HSD test).

**Table 3:** The morphometric data (average ± standard deviation) round goby from Dąbie Lake, Szczecin Lagoon, and Puck Bay (F—female; M—male).

Sampling Place	Sex of Fish	*N*	Total Length cm	Individual Weight g
Dąbie Lake	F	8	15.00 ± 0.55 ^a^	72.41 ± 13.05 ^a^
	M	8	16.70 ± 0.87 ^a^	80.98 ± 9.20 ^a^
	Together	16	15.88 ± 1.11 ^A^	76.69 ± 12.08 ^B^
Szczecin Lagoon	F	8	16.10 ± 0.93 ^a^	69.49 ± 19.26 ^a^
	M	8	16.30 ± 0.86 ^a^	71.27 ± 9.03 ^a^
	Together	16	16.21 ± 0.91 ^A^	70.32 ± 15.38 ^B^
Puck Bay	F	8	16.60 ± 0.98 ^b^	61.82 ± 11.76 ^b^
	M	8	15.20 ± 0.81 ^a^	55.28 ± 9.6 ^a^
	Together	16	16.37 ± 0.93 ^A^	58.55 ± 11.22 ^A^
Average	F	24	15.57 ± 0.97 ^a^	67.91 ± 16.04 ^a^
	M	24	16.07 ± 1.08 ^a^	69.18 ± 14.26 ^a^
	Together	48	15.82 ± 1.05	68.54 ± 15.03

^A,B^—the same indexes in the rows for together indicate no significant differences (one-way ANOVA, post-hoc Tukey HSD, *p* < 0.05). ^a,b^—the same indexes in the rows for female (F) and male (M) (separately for each water station) indicate no significant differences (one-way ANOVA, post-hoc Tukey HSD, *p* < 0.05).

**Table 4:** Comparison of As and Se (average ± standard deviation; concentration in μg kg^−1^) between the studied organs of round goby from Dąbie Lake, Szczecin Lagoon, and Puck Bay.

Elements	Sampling Place	Muscle μg kg^−1^	Liver	Gonads	Gills	Total Average
As	Dąbie Lake	34.7 ± 9.7 ^B^	82.2 ± 16.4 ^C^	55.7 ± 14.1 ^B^	31.3 ± 7.4 ^B^	36.9 ± 23.1
	Szczecin Lagoon	22.1 ± 5.5 ^A^	55.5 ± 17.1 ^B^	50.0 ± 14.51 ^B^	10.6 ± 3.1 ^A^	
	Puck Bay	18.5 ± 4.2 ^A^	38.8 ± 10.9 ^A^	36.4 ±10.5 ^A^	7.3 ± 2.2 ^A^	
	Average	25.1 ± 9.7	58.8 ± 23.3	47.4 ± 15.2	16.4 ± 11.7	
Se	Dąbie Lake	170.3 ± 48.8 ^A^	528.6 ± 183.9 ^B^	671.4 ± 104.3 ^A^	267.4 ± 67.1 ^B^	348.1 ± 219.3
	Szczecin Lagoon	161.0 ±41.9 ^A^	440.4 ± 96.9 ^B^	632.6 ±99.7 ^A^	182.5 ± 29.9 ^A^	
	Puck Bay	153.0 ± 27.9 ^A^	133.2 ± 24.1 ^A^	601.7 ± 124.7 ^A^	235.6 ± 35.3 ^B^	
	Average	161.4 ± 40.3	367.4 ± 208.1	635.3 ± 111.5	228.5 ± 58.1	

^A,B,C^—the same indexes in the rows (separately for As and Se) indicate no significant differences (one-way ANOVA, post-hoc Tukey HSD, *p* < 0.05).

**Table 5:** The mean percentage of inorganic As and Se per 100 g portion of round goby muscles from Dąbie Lake, Szczecin Lagoon, and Puck Bay, considering the dietary reference intakes (DRI) set per day after Baars et al. [50] (a), FAO WHO [21] (b), Salahinejad and Aflaki [51] (c), Otten et al. [35] (d), and EFSA [4] (e); (TDI—tolerable daily intake; PTWI—provisional tolerable weekly intake, ADI—acceptable daily intake, RDA—recommended dietary allowance; UL—tolerable upper intake level). TDI and PTWI were calculated for adults with a body weight of 70 kg.

Elements	DRI	mg Day^−1^	%DRI	Szczecin Lagoon	Puck Bay	Average
			Dąbie Lake			
As *	TDI ^a^	0.07	0.50	0.32	0.26	0.36
	PTWI ^b^	0.15	0.23	0.15	0.12	0.17
	ADI ^c^	0.13	0.30	0.17	0.14	0.20
Se	RDA ^d^	0.055	31.0	29.3	27.8	29.4
	UL ^d^	0.400	4.3	4.0	3.8	4.03
	UL ^e^	0.255	6.7	6.3	6.0	6.33

* Arsenic toxicity was determined based on the assumption that inorganic As constitutes 10% of the total As content in fish muscles [2,7].

**Table 6:** Health risk analysis of inorganic As and Se: estimated daily intake (EDI) and comparison with reference dose (%RfD), target hazard quotient (THQ), and carcinogenic risk (CR) for the mean concentration of elements in the fish muscle from Dąbie Lake, Szczecin Lagoon, and Puck Bay.

Elements	Sampling Place	RfD ^a^ mg kg^−1^ Day^−1^	EDI mg kg^−1^ Day^−1^	%RfD	THQ	CSF ^a^ mg kg^−1^ Day^−1^	CR ^b^ mg kg^−1^ Day^−1^
As	Dąbie Lake	3.00 × 10^−4^	1.49 × 10^−5^	4.96	4.96 × 10^−2^	1.5	2.23 × 10^−6^
	Szczecin Lagoon		9.47 × 10^−6^	3.16	3.16 × 10^−2^		1.42 × 10^−6^
	Puck Bay		7.93 × 10^−6^	2.64	2.64 × 10^−2^		1.19 × 10^−6^
Average			1.08 × 10^−5^	3.59	3.59 × 10^−2^		1.61 × 10^−6^
Se	Dąbie Lake	5.00 × 10^−3^	7.30 × 10^−5^	1.46	1.46 × 10^−2^	-	-
	Szczecin Lagoon		6.90 × 10^−5^	1.38	1.38 × 10^−2^		-
	Puck Bay		6.56 × 10^−5^	1.31	1.31 × 10^−2^		-
Average			6.92 × 10^−5^	1.38	1.38 × 10^−2^		-

^a^ RfD (chronic oral reference dose) and CSF (cancer slope factor) are taken from the US EPA [47]. ^b^ CR was calculated based on the assumption that inorganic As constitutes 10% of the total As content in fish muscles [2,7,55].

## Data Availability

The original contributions presented in the study are included in the article, further inquiries can be directed to the corresponding author.
